# Eculizumab as a treatment for C3 glomerulopathy: a single-center retrospective study

**DOI:** 10.1186/s12882-023-03058-9

**Published:** 2023-01-11

**Authors:** Thomas Welte, Frederic Arnold, Lukas Westermann, Felix A. Rottmann, Martin J. Hug, Elke Neumann-Haefelin, Athina Ganner

**Affiliations:** 1 Department of Nephrology, Medical Center, Faculty of Medicine, University of Freiburg, Freiburg, Germany; 2 Institute for Microbiology and Hygiene, Medical Center, Faculty of Medicine, University of Freiburg, Freiburg, Germany; 3 Pharmacy, Medical Center, Faculty of Medicine, University of Freiburg, Freiburg, Germany

**Keywords:** C3 glomerulopathy, C3 glomerulonephritis, Dense deposit disease, Complement, Eculizumab

## Abstract

**Background:**

C3 Glomerulopathy (C3G) is a rare glomerular disease caused by dysregulation of the complement pathway. Based on its pathophysiology, treatment with the monoclonal antibody eculizumab targeting complement C5 may be a therapeutic option. Due to the rarity of the disease, observational data on the clinical response to eculizumab treatment is scarce.

**Methods:**

Fourteen patients (8 female, 57%) treated for C3 glomerulopathy at the medical center of the University of Freiburg between 2013 and 2022 were included. Subjects underwent biopsy before enrollment. Histopathology, clinical data, and response to eculizumab treatment were analyzed. Key parameters to determine the primary outcome were changes of estimated glomerular filtration rate (eGFR) over time. Positive outcome was defined as > 30% increase, stable outcome as ±30%, negative outcome as decrease > 30% of eGFR.

**Results:**

Eleven patients (78.8%) were treated with eculizumab, three received standard of care (SoC, 27.2%). Median follow-up time was 68 months (IQR: 45–98 months).

Median eculizumab treatment duration was 10 months (IQR 5–46 months). After eculizumab treatment, five patients showed a stable outcome, six patients showed a negative outcome. Among patients receiving SoC, one patient showed a stable outcome, two patients showed a negative outcome.

**Conclusions:**

The benefit of eculizumab in chronic progressive C3 glomerulopathy is limited.

**Supplementary Information:**

The online version contains supplementary material available at 10.1186/s12882-023-03058-9.

## Introduction

C3 glomerulopathy (C3G) is a term adopted to define a group of kidney diseases defined by underlying dysregulation of the alternative pathway of the complement system [1]. C3G is characterized by prominent glomerular deposition of complement C3, either isolated or at least two orders of magnitude greater than other immune reactants [2], and histo-pathological features of membranoproliferative glomerulonephritis (MPGN). Current literature defines two subtypes of C3G spectrum diseases: Dense deposit disease (DDD), characterized by electron-dense deposits within the glomerular basement membrane (GBM), and C3 glomerulonephritis (C3GN), characterized by subendothelial or mesangial deposits.

The disease is caused by activation of the alternative pathway of the complement system, driven by both genetic or acquired defects of complement proteins or cofactors. For example, mutations in *C3, CFB, CFH, CFI, CFHR1–5*, and autoantibodies against CFH, CFB, C3bBb (C3 nephritic factor), often dysregulating C3 convertase activity, are commonly found in patients diagnosed with C3G [3–6].

C3G is a rare disease with an estimated incidence of 1–3 cases per million [7]. Patients present with varying levels of proteinuria and hematuria. Few studies report spontaneous remission [8], but up to 50% of cases progress to end-stage renal disease (ESRD) within a decade after diagnosis. Recurrence rates in allografts amount to up to 60% [9, 10]. Thus, renal prognosis is poor. Age > 16 years, DDD subtype, and crescentic glomerulonephritis (GN) were identified as predictors for ESRD in a cohort study including 80 individuals [7].

In contrast to immune-complex mediated MPGN, where treatment focuses on the underlying diseases, such as chronic infections, autoimmune diseases, or cancer, there are no established treatment options for C3G [11]. Treatment with immunosuppression (calcineurin inhibitors, cyclophosphamide, mycophenolate mofetil, or rituximab [10, 12–15], plasma infusion [16], or plasmapheresis [17] report inconsistent results in small patient cohorts.

Based on the role of the complement system in the etiology of C3G, complement-modulating drugs constitute promising treatment options. Eculizumab, a humanized monoclonal antibody against complement C5, blocks the formation of (sC5b-9), which binds to a membrane as MAC.

An open-label, proof-of-concept, efficacy and safety study, and multiple case series show mixed results, with treatment response mainly observed in crescentic glomerulonephritis [14, 18–33]. As clinical follow-up of published cases is generally short, no conclusions about long-term outcomes of patients treated with eculizumab can be drawn.

Here, we present pathological and clinical features of all patients with C3G treated at the department of Nephrology, University of Freiburg, Germany over the last 20 years. Fourteen patients with C3 glomerulopathy were identified, 11 of which were treated with eculizumab. Hence, we present valuable data on renal outcomes of a comparatively large cohort of C3G cases.

## Methods

We screened medical records of C3G patients treated between May 2002 and May 2022 at the department of Nephrology, University of Freiburg, Germany. Inclusion criteria were age > 18 years at the time of study initiation and histopathological diagnosis of C3G.

Age, sex, information on current medication, serum creatinine levels, urine protein to creatinine ratio (UPCR), serum albumin levels, and hematuria were obtained from electronic patient records. Hematuria was analyzed semi-quantitatively by dipstick measurement. Reference ranges were defined from 0 (no hematuria) to 5 (maximum hematuria). Thus, outcome measures for hematuria must be interpreted with caution. Additionally, data from kidney biopsy reports, and - if available - genetic and immunological test results were extracted. All biopsies were evaluated according to current diagnostic guidelines [1]. Of note, genetic and immunological testing was done non-standardized over a long period of time. If available, the pathophysiological relevance of the genetic findings are discussed for each individual case in the results section. Data from patients receiving dialysis while receiving eculizumab therapy were omitted. In these cases, the last test results before hemodialysis initiation were used. For patients receiving eculizumab, treatment initiation was set as timepoint zero. In patients receiving SoC, the time of first visit was set as timepoint zero.

Outcomes were either measured two months after treatment initiation with eculizumab treatment (early outcomes), or at the end of treatment (late outcomes). Outcome measurements were change of kidney function, quantified by estimated glomerular filtration rate (eGFR). EGFR was calculated using the Chronic Kidney Disease Epidemiology Collaboration (CKD-EPI) equation [34]. Secondary outcomes were change of serum albumin, proteinuria (urine protein to creatinine ratio [UPCR]), and hematuria.

Positive outcome for the variables eGFR and serum albumin was defined as > 30% increase, stable outcome as ±30%, negative outcome as decrease > 30% relative to eGFR and serum albumin measured at the first day of eculizumab treatment. For proteinuria and hematuria, positive outcome was defined as decrease > 30%, stable outcome ±30%, negative outcome as > 30% increase of respective variables relative to the values measured at the first day of eculizumab treatment.

All patients gave informed consent for genetic testing, eculizumab, or alternative immunosuppressive treatment. Eculizumab was given according to aHUS standard treatment protocol [35] as follows: Weekly administration of 900 mg (week 1–4), followed by 1200 mg every other week (from week 5). Eculizumab levels were monitored at physician’s discretion. In patients with trough eculizumab levels < 200 μg/ml, the eculizumab treatment protocol was amended to 900 mg weekly doses. Serum creatinine, urinary protein levels, serum albumin, and hematuria were monitored regularly during routine visits.

Statistical analysis and data visualization were performed using R 4.2.0 statistical software. If not stated otherwise, data are presented as frequency and percentage, or median and interquartile ranges. The following statistical tests were performed: For categorical data, a Pearson’s chi square test was performed, for numeric variables, a group F-test with ANOVA was performed. All analyses with *P* < 0.05 were considered statistically significant.

## Results

### Patient characteristics

Fourteen patients were included in the analysis. Cases C3G1-C3G7 were reported previously [32], and are included in this analysis with extended follow-up. Table 1 and Table S1 summarize the patient’s characteristics. C3G was diagnosed histopathologically on the recommendations of the consensus conference for C3 glomerulopathy [1]. Median age at diagnosis was 27 years (IQR 20.8–57.3). Sex distribution was balanced (57.1% female). Six out of 14 patients (42.9%) were kidney transplant patients.

**Table 1:** Baseline patient characteristics

**Patients (n)**	14
female sex (*n*, [%])	8 (57.1)
kidney transplant at diagnosis (*n*, [%])	6 (42.9)
age at diagnosis (years, [IQR])	27.0 (20.75–57.25)
time diagnosis to eculizumab (months, [IQR])^a^	11.0 (4.5–105.0)
eGFR at treatment start (mL/min/1.73 m^2^, [IQR])	40.59 (31.39–72.48)
UPCR at treatment start (g/g [IQR])	2.51 (0.75–5.21)
serum albumin at treatment start (g/dl, [IQR])	3.73 (3.08–4.21)
urine blood at treatment start (dipstick^b^, 0–5, [IQR])	3.0 (3.0–4.0)
eculizumab treatment duration (months, [IQR])	10.0 (5.0–52.5)
**Histopathology findings**	
glomerula (*n*, [IQR])	15.5 (12.0–20.0)
global sclerosis (%, [IQR])	7 (5–25)
partial sclerosis (%, [IQR])	0 (0–0)
IF/TA (%, [IQR])	10 (5–30)
crescents (%, [IQR])	0 (0–0)
mesangial proliferation (*n*, [%])	13 (92.9)
leukocyte infiltration (0–3, [IQR])	1 (0–1)
**Immunohistochemistry findings**^**c**^	
C3 (0–3, [IQR])	3 (2–3)
C4d (0–3, [IQR])	0 (0–0)
C5b-9 (0–3, [IQR])	1 (1–2)
IgA (0–3, [IQR])	0 (0–1)
IgG (0–3, [IQR])	0 (0–1)
IgM (0–3, [IQR])	1 (1–2)
**Electron microscopy findings**	
DDD typical deposits (n, [%])	4 (30.8)

EGFR, UPCR, hematuria, serum albumin levels are median values and interquartile ranges in the first month of eculizumab treatment. ^a^ Time from diagnosis to eculizumab initiation indicates the time from first diagnosis to first eculizumab treatment. ^b^ hematuria measured by urine dipstick (scale from 0 to 5). ^c^ Data are graded on a range from 0 to 3 based on staining intensity

*Abbreviation*: *IQR* Interquartile range

First diagnosis was C3G in most cases, with the following exceptions: In patients C3G1 and C3G9, MPGN type I was diagnosed prior to transplantation. In these cases, biopsies after kidney transplantation revealed C3G and the time from diagnosis indicates the time since biopsy-proven C3G. Kidney biopsies showed moderate interstitial fibrosis and tubular atrophy (10% [IQR 5–30%]), with one case of crescentic glomerulonephritis (case C3G14). All but one patient presented with mesangial proliferation characteristic for MPGN. As expected, immunohistochemistry in kidney biopsies showed prominent C3 deposition, with no or weak staining for IgG, IgA, and IgM. Electron microscopy detected dense deposits in four out of 14 patients (30.8%). Inflammatory cells were either absent, or weakly detected in most cases (11 out of 14 [78.5%]; Table 1, Table S1).

Autoantibodies against C3 convertase (C3NeF), C3B, CFB, CFH were detected in six out of 14 (42.9%) of cases (C3G1–4, C3G9, and C3G14; Table S1).

Genetic variants detected in cases C3G1, C3G5, and C3G6 have been discussed in a previous report [32]. Patient C3G12 presented with heterozygous mutations in *C3* (c.2531A > G; p.[Q844R]) and *C9* (c.1677del; p.[K559Nfs*49]) of potential pathophysiological relevance [36]. Patient C3G11 presented with two potentially pathogenic variants (het *CFH-H1* haplotype, het c.193A > C [K65Q] [37].

All patients received treatment with angiotensin converting enzyme inhibitors (ACEI), or angiotensin receptor blockers (ARB) for blood pressure control and reduction of proteinuria (SoC treatment).

Three out of 14 patients (21.4%) received only SoC, with one patient receiving treatment with mycophenolate mofetil (MMF). All other patients (11 of 14; 78.6%) received eculizumab. Among these patients, eight (72.7%) received additional immunosuppression parallel to eculizumab treatment. Among these were six kidney transplant recipients receiving immunosuppression with tacrolimus, mmf, and corticosteroids. Details on treatment intervals and substances for each case can be found in Figs. S1 and S2.

Median time from diagnosis to eculizumab treatment was 11 months (IQR: 4.5–105.0). At treatment initiation, patients presented with moderately impaired eGFR (40.6 ml/min/1.73 m^2^ (IQR: 31.4–72.5), close to nephrotic range proteinuria (UPCR: 2.5 g/g [IQR 0.8–5.2 g/g], and hematuria. Median follow-up time was 68 months (IQR: 45–98 months). Median eculizumab treatment duration was 10 months (IQR 5–46 months).

All patients received meningococcal vaccinations prior to eculizumab initiation. One case of severe meningococcal sepsis with Waterhouse-Friderichsen syndrome was reported in patient C3G3 with missing meningococcal booster vaccination > 5 years after base immunization. In this patient, treatment had to be discontinued for 1 month, leading to a rapid eGFR decline, followed by stabilization after eculizumab re-initiation (Fig. S1). In all other cases, no severe side effects were reported during eculizumab treatment.

### Outcomes

Outcomes were analyzed two months after treatment initiation (early outcome), and at the end of treatment (late outcome). One patient had to be omitted in early outcome analysis, as laboratory data were not available for this timepoint. Table S2 summarizes raw data and outcomes for each patient.

**Fig. 1:**
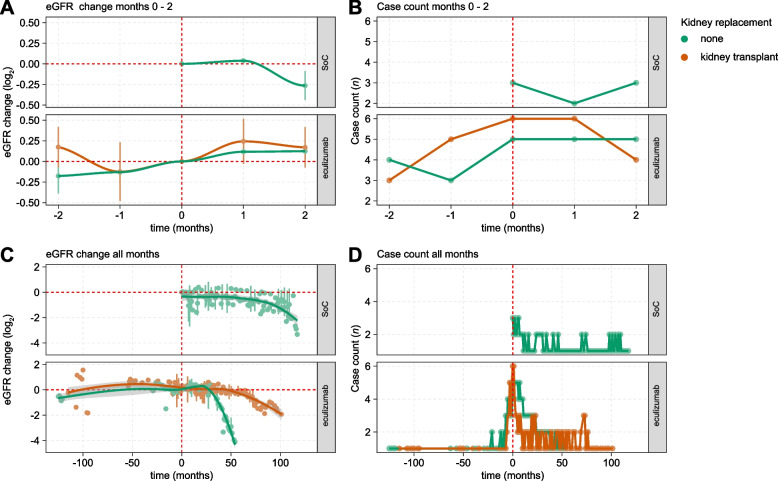
Response to eculizumab treatment. **A**, **C** Line graphs showing eGFR change in log_2_ scale (y-axis) relative to month zero. X-axis indicates time in months. Upper facet shows patients receiving standard of care (SoC) treatment, lower facet shows patients receiving eculizumab treatment. Dots indicate median eGFR values for all available cases at respective months. Vertical lines indicate standard deviations. Smooth line indicates a generalized additive model fitted to the data. Grey areas indicate 95% confidence intervals for the model. **B**, **D** Line graph indicating count of cases (y-axis) with available eGFR data for each timepoint (x-axis; time in months). Patient data was grouped and colored in cases with native kidneys, or kidney allografts. Coloring is indicated in the legend to the right

After 2 months, 12 out of 13 analyzed patients (92.3%) had a stable eGFR while this parameter had improved in one patient (7.7%). A decrease in eGFR was not detected in any patient. Notably, we observe a trend for improved eGFR in the eculizumab group in comparison to patients treated SoC in both native kidneys and renal allografts (Fig. 1A, B), indicating possible short-term efficacy of eculizumab in our cohort.

At treatment end, 8 patients (57.1%) had a negative, 6 patients (42.9%) a stable, and no patient an improved outcome. Patients with negative outcome had longer treatment time in comparison to patients with stable outcome (26 vs 3 months), indicating an increasing risk of eGFR deterioration in longer treatment intervals. EGFR, UPCR, serum albumin levels, and urine blood levels at treatment start and at two months were similar between the groups. Patients with negative outcomes at treatment end were older than patients with stable outcome (median 41.5 vs 24 years) and had longer timespans to treatment initiation (median 49.5 vs 7 months). Notably, patients receiving eculizumab treatment were not overrepresented in stable vs negative outcomes at treatment end (75% vs 83%). In contrast to a previous study [33], we observe no correlation between glomerular scarring and renal outcomes, which might be explained by uneven numbers of glomerula in kidney biopsies between the groups. Staining intensities for C3 in kidney biopsies were similar between the groups (Table 2).

**Table 2:** Outcomes

	Outcome eGFR at 2 months					Outcome eGFR at treatment end				
Variable	***n***	positive^**a**^	***n***	stable^**a**^	***p***^**b**^	***n***	negative^**a**^	***n***	stable^**a**^	***p***^**b**^
**Sex**	1		12		0.94	8		6		0.31
F	0	0%	7	58%		6	75%	2	33%	
M	1	100%	5	42%		2	25%	4	67%	
**Native/Transplant**	1		12		0.81	8		6		1
Native	0	0%	8	67%		5	62%	3	50%	
Transplant	1	100%	4	33%		3	38%	3	50%	
**Eculizumab**	1		12		1	8		6		1
No	0	0%	3	25%		2	25%	1	17%	
Yes	1	100%	9	75%		6	75%	5	83%	
Age at diagnosis (years)	1	58	12	25.5 (18.8–43.8)	0.22	8	41.5 (23.3–59,5)	6	24.0 (23.0–40.0)	0.32
Time diagnosis to eculizumab (months) ^c^	1	2	9	60 (7–122)	0.35	6	49.5 (8.0–118.0)	5	7 (2.0–60.0)	0.52
Eculizumab treatment duration (months)	1	2	12	2		8	26.0 (5.2–65.8)	6	3.0 (3.0–7.0)	0.08
**Secondary outcomes**										
**UPCR at 2 months**	0		6			4		2		0.22
negative	0	NA	2	33%		2	50%	0	0%	
positive	0	NA	1	17%		1	25%	0	0%	
stable	0	NA	3	50%		1	25%	2	100%	
**Serum albumin at 2 months**	0		4			2		2		1
positive	0	NA	1	25%		1	50%	0	0%	
stable	0	NA	3	75%		1	50%	2	100%	
**Urine blood at 2 months**	0		4			3		1		1
negative	0	NA	1	25%		1	33%	0	0%	
stable	0	NA	3	75%		2	67%	1	100%	
**UPCR at treatment end**	1		8		0.14	4		5		0.89
negative	0	0%	4	50%		2	50%	2	40%	
positive	1	100%	1	12%		1	25%	1	20%	
stable	0	0%	3	38%		1	25%	2	40%	
**Serum albumin at treatment end**	1		8		1	5		4		0.91
negative	0	0%	1	12%		0	0%	1	25%	
stable	1	100%	7	88%		5	100%	3	75%	
**Urine blood at treatment end**	1		6		1	3		4		0.74
positive	0	0%	3	50%		2	67%	1	25%	
stable	1	100%	3	50%		1	33%	3	75%	
**Laboratory parameters**										
**treatment start**										
eGFR (ml/min/1.73 m^2^)	1	13.6	12	45.6 (38.2–79.9)	0.16	8	45.6 (29.4–79.9)	6	40.5 (34.9–41.6)	0.48
UPCR (g/g)	1	6.06	9	2.29 (0.3–4.4)	0.42	6	3.4 (2.3–4.3)	5	1.2 (0.3–6.1)	0.93
Serum albumin (g/dl)	1	3.95	8	3.38 (2.9–4.0)	0.56	6	3.38 (3.1–4.1)	4	3.9 (3.5–4.1)	0.6
Urine blood dipstick (0–5)	1	5	7	3 (2.8–3.5)	0.18	5	3 (3–4)	4	3.25 (1.9–4.3)	0.52
**at 2 months treatment**										
eGFR (mL/min/1.73 m^2^)	1	20.8	12	50.6 (41.2–63.8)	0.24	7	55.9 (39.1–65.1)	6	43.8 (37.8–45.1)	0.41
UPCR (g/g)	0	NA	6	2.54 (1.5–2.8)		4	2.54 (1.88–2.7)	2	5.01 (3.08–6.94)	0.3
Serum albumin (g/dl)	0	NA	4	3.75 (3.3–4.0)		2	3.75 (3.7–3.8)	2	3.35 (2.8–3.9)	0.76
Urine blood dipstick (0–5)	0	NA	5	4 (3.0–4.0)		3	4 (3–4)	2	3.5 (3.3–3.8)	0.87
**at tretment end**										
eGFR (mL/min/1.73 m^2^)	1	16.1	12	33.2 (13.2–41.3)	0.45	8	12.9 (9.6–26.7)	6	38 (32.9–42,6)	0.02
UPCR (g/g)	1	3.52	11	2.25 (0.6–5.8)	0.92	6	3.23 (1.4–6.5)	6	2.23 (0.1–3.5)	0.57
Serum albumin (g/dl)	1	3.7	9	3.2 (2.7–4.3)	0.7	6	3.1 (2.8–4.0)	4	3.65 (3.2–4.1)	0.57
Urine blood dipstick (0–5)	1	5	11	2 (1.5–3.0)	0.11	6	2 (1.3–2.8)	6	2.5 (2–4)	0.63
**Histopathology findings**										
**Glomerula (*n*)**	1	8	12	17.5 (14.0–21.3)	0.39	8	15 (10.3–16.8)	6	22.5 (15–25)	0.06
Global sclerosis (%)	1	37.5	11	7 (3.5–20.5)	0.42	8	6.7 (3.75–10.75)	5	37.5 (7–56)	0.04
Partial sclerosis (%)	1	12.5	11	0 (0–0)	0.9	8	0 (0–1.6)	5	0 (0–0)	0.52
IF/TA (%)	1	15	9	10 (5.0–30.0)	1	7	10 (5–30)	4	12.5 (8.8–21.3)	0.85
Crescents (%)	1	0	12	0 (0–0)	0.79	8	0	6	0	0.26
**Mesangial proliferation**	1		12		1	8		6		1
neg	0	0%	1	8%		1	12%	0	0%	
pos	1	100%	11	92%		7	88%	6	100%	
**DDD typical deposits**	1		12		0.67	8		6		1
neg	0	0%	9	75%		6	75%	4	67%	
pos	1	100%	3	25%		2	25%	2	33%	
**Leukocyte infiltration (0–3)**^**d**^	1	1	12	1 (0–1.3)	0.85	8	1 (0–1)	6	1 (0–1)	0.57
**C3 (0–3)**^**e**^	1	3	12	2.5 (2–3)	0.38	8	3 (2–3)	6	2.5 (2–3)	0.67
**C4d (0–3)**^**e**^	1	0	2	0 (0–0)		2	0 (0–0)	2	0 (0–0)	
**C5b-9 (0–3)**^**e**^	1	1	10	1 (1–2)	0.68	7	1 (1–2)	5	1 (1–1.3)	0.26
**IgA (0–3)**^**e**^	1	0	12	0.5 (0–1)	0.38	7	1 (0–1)	6	0 (0–1)	0.43
**IgG (0–3)**^**e**^	1	0	12	0 (0–1)	0.49	8	1 (0–1)	6	0 (0–0)	0.43
**IgM (0–3)**^**e**^	1	1	12	1 (1–2)	0.53	8	1 (1–1.3)	6	1.5 (1–2)	0.37

^a^ Discrete variables: Number, n (%); continuous variables: Median (IQR).. ^b^*P* value for discrete variables: Pearson’s chi square test; for continuous variables: group F-test with ANOVA. ^c^ Time from diagnosis to eculizumab initiation indicates the time from first diagnosis to first eculizumab treatment. ^d^ Leukocyte infiltration was graded on a scale from 0 to 3. ^d^Data are graded on a scale from 0 to 3 based on staining intensity. ^e^ Data are graded on a range from 0 to 3 based on staining intensity

*Abbreviations*: *C3* complement C3, *C4d* complement 4d, *C5b-9* complement C5b-9, *eGFR* estimated glomerular filtration rate, calculated by CKD-EPI formula, *F* Female, *IF/TA* Interstitial fibrosis and tubular atrophy, *M* Male, *NA* No data avaliable no data avaliable, *Native/Transplant* Native kidney/ kidney allograft, *SD* Standard deviation, *UPCR* Urine protein creatinine ratio

Having observed no significant overrepresentation of eculizumab treatment in stable vs negative late outcomes, we aimed to analyze the effect of eculizumab in greater detail. Direct comparison of patient groups with both native kidneys and renal allografts treated with eculizumab and patients receiving SoC showed no improved eGFR at treatment end (Fig. 1C, D), with a trend towards lower relative eGFR in the eculizumab group. This effect is likely driven by low patient count in later timepoints. Of note, cases receiving SoC had both significantly higher eGFR at treatment start (SoC vs eculizumab treatment: median 81.2 vs 39.3 ml/min/1.73 m^2^, *p* = 0.028), at 2 months (SoC vs eculizumab treatment: median 67.7 vs 43.8 ml/min/1.73 m^2^, *p* = 0.288), and at late timepoints (SoC vs eculizumab treatment: median 40.9 vs 22.5 ml/min/1.73 m^2^, *p* = 0.308), potentially biasing the results (Table S3). However, relative eGFR loss was similar between the groups (Fig. 1A, C).

Hence, although there might be a slight short-term benefit for eculizumab treatment in our cohort, we neither observe improved long-term outcomes in patients treated with eculizumab, nor overrepresentation of patients receiving eculizumab treatment in patients in stable vs negative outcomes. In conclusion, we do not detect improved or stabilized kidney function in patients treated with eculizumab for C3G.

## Discussion

Although we observe a minor trend for improved kidney function early after eculizumab treatment initiation, we fail to observe improved outcomes in later timepoints. This is not unexpected, as eculizumab targets assembly of the MAC complex in the final cascade of the complement pathway. While this mode of action may be effective in acute glomerular inflammation, exemplified by the improved early response to eculizumab reported by previous studies [23, 28, 29, 33, 38], and the renal response to eculizumab re-initiation in patient C3G3, it has no effect on C3 convertase dysregulation and does not inhibit glomerular complement C3 deposition in animal models [39]. This might explain the mixed treatment efficacy in C3G reported here and elsewhere [14, 18–31]. Disease progression is vastly heterogenous in our study population, ranging from gradual decline in kidney function (cases C2G2, 3, 5–7, 9, 10) with eculizumab treatment, to relapsing disease upon discontinuation of eculizumab in case C3G1 and C3G3, to stable disease (case C3G14), or declining kidney function (case C3G13) with supportive therapy or after eculizumab discontinuation (case C3G4).

Collectively, these data demonstrate that efficacy of eculizumab treatment in C3G is questionable. While we stress that eculizumab treatment can be successful in patients with glomerular inflammation, its efficacy in slowly progressive disease seems to be limited. As observed here (cases C3G1, 2, 3, 5, 8, 9, 11) and elsewhere, C3 glomerulopathy is a systemic disease with relapse rates of up to 60% in allografts [9, 10]. Hence, close clinical monitoring is crucial.

We and others [7, 33, 40] observe age at diagnosis to be a risk factor for disease progression.

Other disease parameters, such as proteinuria, serum albumin levels, and hematuria were difficult to interpret due to rare testing. Thus, standardized follow-up in larger cohorts will be necessary to obtain conclusive results. Of note, hematuria was evaluated semi-quantitatively using dipstick measures. Thus, results for hematuria outcomes should be interpreted with caution. As nephrotic range proteinuria commonly leads to accelerated loss of immunoglobulins [41], it may also lead to accelerated elimination of eculizumab. Thus, eculizumab levels should be quantified regularly to avoid underdosing in these patients.

This study has several limitations, the major being the retrospective character and the lack of standardized follow-up, and a heterogenous population with both native kidneys and kidney grafts, as well as a small control cohort receiving SoC. Additionally, the multitude of therapeutic approaches prior to, or in parallel with eculizumab treatment complicate the interpretation. We underline the need to prospectively collect data of patients with C3G.

In conclusion, C5 blockage by eculizumab might be a therapeutic option for acute progressive disease with glomerular inflammation, but its efficacy in chronic progressive C3 glomerulopathy seems to be limited. Novel therapeutic agents targeting the C3 convertase are currently tested in clinical studies. These new therapies might transform the treatment of this complex rare disease.

## Supplementary Information

**Additional file 1: Supplementary Table S1.** Patients` characteristics. **Supplementary Table S2.** Overview of individual outcomes. **Supplementary Table S3.** Overview of cohort outcomes grouped by eculizumab treatment. **Supplementary Fig. S1.** Course of eGFR and UPCR for individual cases. **Supplementary Fig. S2.** Course of serum albumin and urine blood for individual cases.

## Data Availability

The datasets analyzed in the current study are not publicly available to ensure privacy of research participants and comply with regulations of the ethics approval. The datasets used and/or analysed during the current study are available from the corresponding author on reasonable request.

## Abbreviations

ACEI: Angiotensin converting enzyme inhibitors
aHUS: Atypical hemolytic uremic syndrome
ARB: Angiotensin receptor blockers
C3G: C3 glomerulopathy
C3NeF: C3 nephritic factor
CyA: Cyclosporine A
DDD: Dense deposit disease
del: Deletion
eGFR: Estimated glomerular filtration rate
ESRD: End stage renal disease
ffp: Fresh frozen plasma
het: Heterozygous
hom: Homozygous
IF/TA: Interstitial fibrosis and tubular atrophy
mmf: Mycophenolate mofetil
MPGN: Membranoproliferative glomerulonephritis
NA: No data avaliable
pex: Plasmapheresis
SoC: Standard of care
UPCR: Urinary protein to serum protein ratio
