# Supplementary material for: Eculizumab as a treatment for C3 glomerulopathy: a single-center retrospective study
Source: BMC Nephrol. 2023 Jan 11;24:8. doi: 10.1186/s12882-023-03058-9 (PMC9832765; doi:10.1186/s12882-023-03058-9)
Supplement: Supplementary file 1 — Additional file 1: Supplementary Table S1. Patients` characteristics. Supplementary Table S2. Overview of individual outcomes. Supplementary Table S3. Overview of cohort outcomes grouped by eculizumab treatment. Supplementary Fig. S1. Course of eGFR and UPCR for individual cases. Supplementary Fig. S2. Course of serum albumin and urine blood for individual cases. [file 12882_2023_3058_MOESM1_ESM.pdf]

Supplementary Material to

Eculizumab as a treatment for C3 glomerulopathy: a single-center retrospective study

Thomas Welte; Frederic Arnold; Lukas Westermann; Felix A. Rottmann; Martin J. Hug; Elke Neumann-Haefelin; Athina Ganner

This PDF file includes:
Supplementary Tables S1 – 3
Supplementary Figures S1 – 2
References to Supplementary Material

ID	C3G 1	C3G 2	C3G 3	C3G 4	C3G 5	C3G 6	C3G 7	C3G 8	C3G 9	C3G 10	C3G 11	C3G 12	C3G 13	C3G 14
**General parameters**														
Sex	M	M	F	F	F	F	F	M	F	F	M	M	M	F
Native/Transplant	Transplant	Transplant	Native	Native	Transplant	Na-tive	Native	Transplant	Transplant	Native	Transplant	Na-tive	Native	Native
Age at diagnosis (years)	20	26	15	40	28	59	14	58	61	55	23	25	68	10
Time diagnosis to Eculizumab (months) [a]	2	144	11	7	88	7	128	2	1	NA	122	60	NA	NA
eGFR at treatment start (mL/min/1.73 m^2)	49.64	39.58	112.65	39.31	30.23	26.95	51.57	13.55	18.53	79.45	34.89	41.60	81.18	82.18
UPCR at treatment start (g/g)	1.20	6.10	2.29	0.30	0.18	4.35	4.19	6.06	2.51	NA	0.08	9.65	NA	NA
Serum albumin at treatment start (g/dl)	NA	1.60	3.15	3.85	NA	3.05	3.60	3.95	4.50	NA	4.50	2.55	4.30	NA
Urine blood at treatment start (0-5) [b]	NA	4.00	NA	4.00	3.00	3.00	3.00	5.00	5.00	NA	0.00	2.50	NA	NA
Follow-up time (months)	43	95	102	61	75	99	63	21	17	122	29	73	53	114
**Kidney biopsy light microscopy findings**														
Glomerula (*n*)	25	20	19	15	3	11	8	8	15	15	20	50	16	36
… Global sclerosis (%)	56	5	16	7	0	9	0	37.5	6.7	6.7	65	2	25	NA
… Partial sclerosis (%)	0	0	0	0	0	91	0	12.5	0	0	0	0	6.25	NA
IF/TA (%)	5	5	10	10	0	30		15	30	5	40	NA	30	NA
Crescents (%)	0	0	0	0	0	0	0	0	0	0	0	0	0	5
Mesangial proliferation	pos	pos	pos	pos	pos	neg	pos	pos	pos	pos	pos	pos	pos	pos
Leukocyte infiltration (0-3) [c]	1	2	0	2	1	0	0	1	1	1	0	0	1	2
**Immunohistochemistry findings**														
C3 (0-3)[d]	2	3	3	2	3	2	2	3	3	3	3	2	2	3
C4d (0-3)[d]	0	NA	NA	NA	0	NA	NA	0	0	NA	NA	NA	NA	NA
C5b-9 (0-3)[d]	1	2	2	1	2	1	NA	1	1	1	2	NA	1	0
IgA (0-3)[d]	0	1	0	1	0	1	1	0	NA	1	0	1	0	0
IgG (0-3)[d]	0	1	1	0	1	0	1	0	1	0	0	2	0	0
IgM (0-3)[d]	1	1	1	2	1	2	1	1	2	1	1	2	1	2
**Electron microscopy findings**														
DDD typical deposits	neg	neg	neg	neg	neg	pos	pos	pos	neg	neg	neg	neg	neg	pos
**Immunology workup**														
C3Bb antibody	pos	pos	pos	pos	NA	NA	NA	NA	NA	NA	NA	NA	NA	pos
C3B antibody	NA	NA	NA	pos	NA	NA	NA	NA	NA	NA	NA	NA	NA	NA
CFB antibody	NA	NA	NA	pos	NA	NA	NA	NA	NA	NA	NA	NA	NA	NA
CFH antibody	NA	NA	pos	NA	NA	NA	NA	NA	pos	NA	NA	NA	NA	NA
**Genetic workup**														
CFH	NA	NA	NA	NA	NA	NA	NA	NA	NA	NA	*het CFH-H1*	NA	NA	NA
CFI	*het c.1322 A>G*	NA	NA	NA	NA	NA	NA	NA	NA	NA	NA	NA	NA	NA
CFB	NA	NA	NA	NA	NA	NA	NA	NA	NA	NA	NA	NA	NA	NA
CFHR1-5	*het del CFHR1,3*	NA	NA	NA	NA	*het CFHR5 c.832 G>A*	NA	NA	NA	NA	*het del CFHR1,3*	*het del CFHR1,3*	NA	NA
MCP	NA	NA	NA	NA	NA	NA	NA	NA	NA	NA	NA	NA	NA	NA
COL4A3	*het c.4484 A>G*	NA	NA	NA	NA	NA	NA	NA	NA	NA	NA	NA	NA	NA
C3	NA	NA	NA	NA	*het c.2203 C>T*	NA	NA	NA	NA	NA	*het c.193 A>C*	*het c.2531 A>G*	NA	NA
C9	NA	NA	NA	NA	NA	NA	NA	NA	NA	NA	NA	*het c1677 delA*	NA	NA

Supplementary Table S1. Patients` characteristics.

EGFR, UPCR, hematuria, serum albumin levels are median values and interquartile ranges in the first month of eculizumab treatment. [a] Time from diagnosis to eculizumab initiation indicates the time from first diagnosis to first eculizumab treatment. [b] Hematuria measured by dipstick. Data are graded on a scale from 0 to 5 based on staining intensity. [c] Leukocyte infiltration was graded on a scale from 0 to 3. [d]Data are graded on a scale from 0 to 3 based on staining intensity. Abbreviations: C3, complement C3; C4d, complement 4d; C5b-9, complement C5b-9; eGFR, estimated glomerular filtration rate, calculated by CKD-EPI formula; F, female; IF/TA, interstitial fibrosis and tubular atrophy; M, male ; NA, no data avaliable; Native/Transplant, native kidney/ kidney allograft; UPCR, urine protein creatinine ratio.

ID	C3G 1	C3G 2	C3G 3	C3G 4	C3G 5	C3G 6	C3G 7	C3G 8	C3G 9	C3G 10	C3G 11	C3G 12	C3G 13	C3G 14
**Treatment start**														
eGFR at treatment start (mL/min/1.73 m^2)	49.64; 32.30	39.58	112.65; 11.19; 29.67	39.31	30.23	26.95	51.57	13.55	18.53	79.45	34.89	41.60	81.18	82.18
UPCR at treatment start (g/g)	1.20	6.10	2.29; 13.67; 0.34	0.30	0.18	4.35	4.19	6.06	2.51	NA	0.08	9.65	NA	NA
Serum albumin at treatment start (g/dl)	NA: NA	1.60	3.15; 2.29; 3.60	3.85	NA	3.05	3.60	3.95	4.50	NA	4.50	2.55	4.30	NA
Urine blood at treatment start (0-5) [a]	NA; 4	4	NA; 2.5; 5	4	3	3	3	5	5	NA	0	3	NA	NA
Eculizumab treatment duration (months)	3; 3	76	42; 12; 29	26	74	10	63	3	7	NA	2	7	NA	NA
**Early outcome**														
eGFR at 2 months (mL/min/1.73 m^2)	54.89; 18.67	46.39	132.46; 25.92; 44.92	45.10	31.83	29.37	55.91	20.80	NA	62.49	37.48	42.46	67.67	81.61
UPCR at 2 months (g/g)	1.15; 5.15	2.66	2.43; 10.8; 0.14	NA	0.23	2.79	NA	NA	NA	NA	NA	8.87	NA	NA
Serum albumin at 2 months (g/dl)	NA; 2.7	3.80	3.7; 2.6; 4.25	NA	NA	NA	NA	NA	NA	NA	4.50	2.20	NA	NA
Urine blood at 2 months (0-5) [a]	4; 3	4	NA; 2; 2	NA	4	2	NA	NA	NA	NA	NA	3	NA	NA
… outcome eGFR at 2 months	stable	stable	stable	stable	stable	stable	stable	positive	NA	stable	stable	stable	stable	stable
… outcome UPCR at 2 months	stable	positive	stable	NA	negative	negative	NA	NA	NA	NA	NA	stable	NA	NA
... outcome serum albumin at 2 months	NA	positive	stable	NA	NA	NA	NA	NA	NA	NA	stable	stable	NA	NA
… outcome at urine blood at 2 months	NA	stable	NA	NA	negative	stable	NA	NA	NA	NA	NA	stable	NA	NA
**Late outcome**														
eGFR at treatment end (mL/min/1.73 m^2)	54.89; 18.67	22.46	39.29; 6.24; 29.67	42.57	13.45	12.41	9.75	16.09	9.21	7.98	32.93	33.45	40.85	68.30
UPCR at treatment end (g/g)	1.15; 5.15	2.25	7.31; NA; 0.57	0.07	NA	4.22	13.3	3.52	NA	1.15	0.14	10.93	0.08	3.32
Serum albumin at treatment end (g/dl)	NA; 2.5	1.95	3.00; 2.75; NA	NA	NA	2.70	3.20	3.70	NA	4.30	4.40	2.60	4.30	3.60
Urine blood at treatment end (0-5) [a]	4; 3	5	3; NA; 2	2	NA	2	2	5	NA	1	0	3	0	2
… outcome GFR at treatment end	stable; negative	negative	negative; negative; stable	stable	negative	negative	negative	stable	negative	negative	stable	stable	negative	stable
… outcome UPCR at treatment end	stable; stable	positive	negative; NA; negative	negative	NA	stable	negative	positive	NA	NA	negative	stable	NA	NA
… outcome serum albumin at treatment end	NA; NA	stable	stable; stable; NA	negative	NA	stable	stable	stable	NA	NA	stable	stable	stable	NA
… outcome urine blood at treatment end	NA; stable	stable	NA; NA; positive	positive	NA	positive	positive	stable	NA	NA	stable	stable	NA	NA

Supplementary Table S2. Overview of individual outcomes.

In case of several eculizumab treatment phases, outcomes are quantified individually for each treatment phase and separated by semicolon. [a] Hematuria measured by dipstick. Data are graded on a scale from 0 to 5 based on staining intensity. Abbreviations: eGFR, estimated glomerular filtration rate, calculated by CKD-EPI formula; F, female; M, male ; NA, no data avaliable; Native/Transplant, native kidney/ kidney allograft; UPCR, urine protein creatinine ratio.

Eculizumab treatment	*n*	No[a]	*n*	Yes[a]	*p*[b]
**primary outcome**					
**Sex**	3		11		1
... F	2	67%	6	55%	
... M	1	33%	5	45%	
**Native/Transplant**	3		11		0.301
... Native	3	100%	5	45%	
... Transplant	0	0%	6	55%	
**outcome GFR at 2 months**	3		10		1
... positive	0	0%	1	10%	
... stable	3	100%	9	90%	
**outcome GFR at treatment end**	3		11		1
... negative	2	67%	6	55%	
... stable	1	33%	5	45%	
Age at Diagnosis (years)	3	55 (32.5 – 61.5)	11	26 (21.5 – 49.0)	0.436
Time diagnosis to eculizumab (months)	0	NA	11	11 (4.5 – 105.0)	
Eculizumab treatment duration (months)	3	0	11	10 (5.0 – 52.5)	0.136
**secondary outcomes**					
**outcome UPCR at 2 months**	0		6		
... negative	0	NA	2	33%	
... positive	0	NA	1	17%	
... stable	0	NA	3	50%	
**UPCR at treatment end**	0		9		
... negative	0	NA	4	44%	
... positive	0	NA	2	22%	
... stable	0	NA	3	33%	
**outcome serum albumin at months**	0		4		
... positive	0	NA	1	25%	
... stable	0	NA	3	75%	
**outcome serum albumin at treatment end**	1		8		1
... negative	0	0%	1	12%	
... stable	1	100%	7	88%	
**outcome urine blood at 2 months**	0		4		
... negative	0	NA	1	25%	
... stable	0	NA	3	75%	
**outcome urine blood at treatment end**	0		7		
... positive	0	NA	3	43%	
... stable	0	NA	4	57%	
**Laboratory parameters**					
**… at treatment start**					
eGFR at treatment start (mL/min/1.73 m^2)	3	81.2 (80.3 – 81.7)	11	39.3 (28.6 – 45.6)	0.028
UPCR at treatment start (g/g)	0	NA	11	2.5 (0.8 – 5.2)	
Serum albumin at treatment start (g/dl)	1	4.3 (4.3 – 4.3)	9	3.6 (3.1 – 4)	0.4
Urine blood at treatment start, dipstick (value 0-5)	0	NA	9	3 (3 – 4)	
**… at 2 months**					
eGFR at 2 months (mL/min/1.73 m^2)	3	67.7 (65.1 – 74.6)	10	43.8 (33.2 – 52.8)	0.288
UPCR at 2 months (g/g)	0	NA	6	2.5 (1.5 – 2.8)	
Serum albumin at 2 months (g/dl)	0	NA	4	3.75 (3.3 – 4.0)	
Urine blood at 2 months, dipstick (value 0-5)	0	NA	5	4 (3 – 4)	
**… at treatment end**					
eGFR at treatmnent end (mL/min/1.73 m^2)	3	40.9 (24.4 – 54.6)	11	22.5 (12.9 – 36.4)	0.308
UPCR at treatment end (g/g)	3	1.2 (0.6 – 2.2)	9	3.5 (1.2 – 7.3)	0.287
Serum albumin at treatment end (g/dl)	3	4.3 (4.0 – 4.3)	7	3.1 (2.7 – 3.9)	0.081
Urine blood at treatment end, dipstick (value 0-5)	3	1 (0.5 – 1.5)	9	3 (2 – 3.8)	0.091
**Histopathology findings**					
**Glomerula (n)**	3	16 (15.5 – 26.0)	11	15 (10.3 – 20.0)	0.572
… Global sclerosis (%)	2	15.9 (11.3 – 20.4)	11	6.9 (4.3 – 21.4)	0.879
… Partial sclerosis (%)	2	3.12	11	0	0.76
**IF/TA (%)**	2	17.5 (11.3 – 23.8)	9	10 (5.0 – 26.3)	0.904
**Crescents (%)**	3	0 (0 – 2.5)	11	0 (0 – 0)	0.051
**Mesangial proliferation**	3		11		1
... neg	0	0%	1	9%	
... pos	3	100%	10	91%	
**DDD typical deposits**	3		11		1
... neg	2	67%	8	73%	
... pos	1	33%	3	27%	
**Leukocyte infiltration (0-3)**[c]	3	1 (1 – 1.5)	11	1 (0 – 1)	0.242
**C3 (0-3)** [c]	3	3 (2.5 – 3)	11	3 (2 – 3)	0.732
**C4d (0-3)** [c]	0	NA	4	0 (0 – 0)	
**C5b-9 (0-3)** [c]	3	1 (0.5 – 1)	9	1 (1 – 2)	0.055
**IgA (0-3)** [c]	3	0 (0 – 0.5)	10	0.5 (0 – 1)	0.646
**IgG (0-3)** [c]	3	0 (0 – 0)	11	0.5 (0 – 1)	0.138
**IgM (0-3)** [c]	3	1 (1 – 1.5)	11	1 (1 – 2)	0.93

Supplementary Table S3. Overview of cohort outcomes grouped by eculizumab treatment.

[a] Discrete variables: Number, n (%); continuous variables: Median (IQR).. [b] *P* value for discrete variables: Pearson's chi square test; for continuous variables: group F-test with Anova. [c] Data are graded on a range from 0 to 3 based on staining intensity. Abbreviations: C3, complement C3; C4d, complement 4d; C5b-9, complement C5b-9; eGFR, estimated glomerular filtration rate, calculated by CKD-EPI formula; F, female; IF/TA, interstitial fibrosis and tubular atrophy; M, male ; NA, no data avaliable; Native/Transplant, native kidney/ kidney allograft; SD, standard deviation; UPCR, urine protein creatinine ratio.

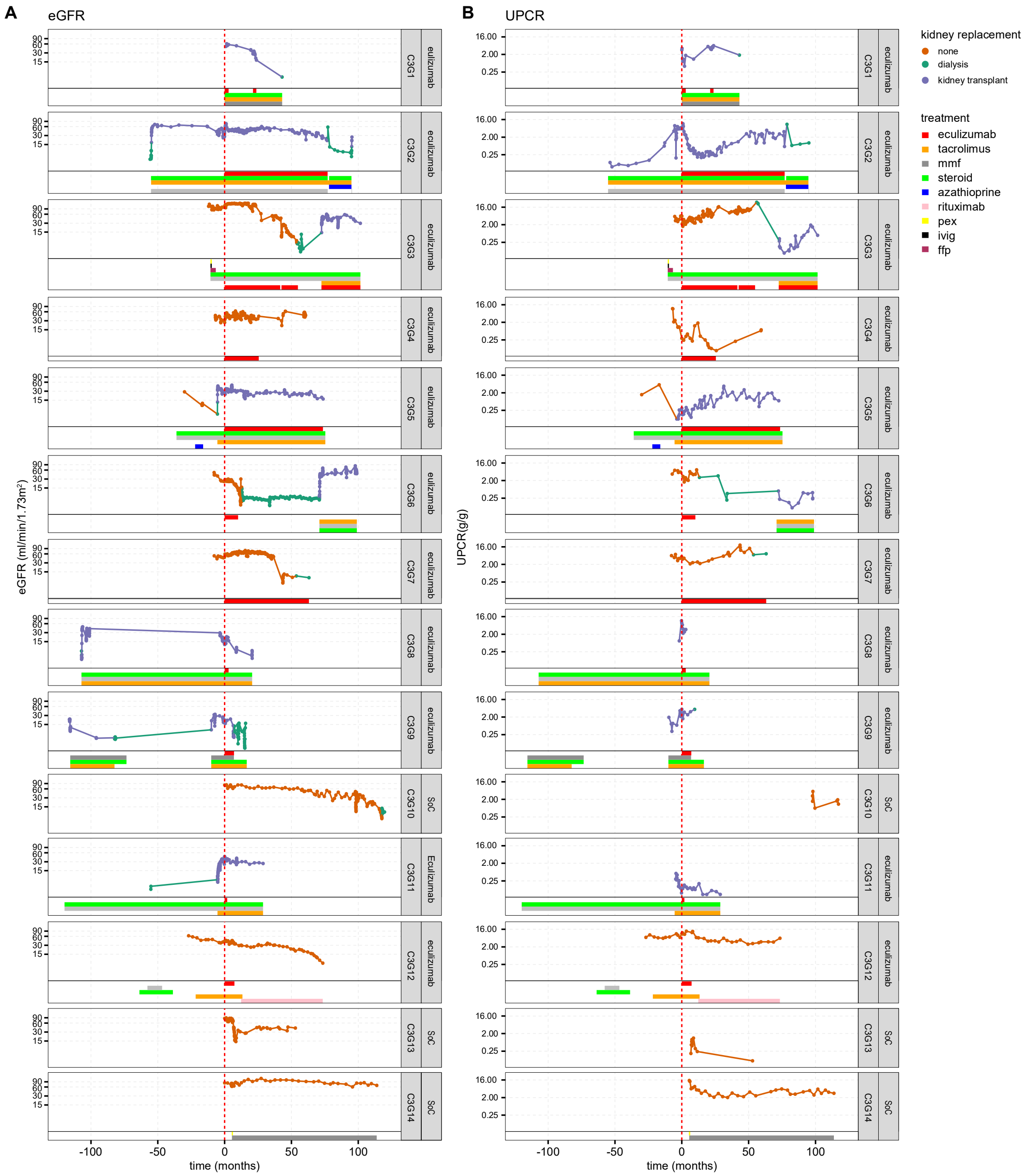

Supplementary Figure S1. Course of eGFR and UPCR for individual cases.
Line graphs indicating eGFR calculated using the CKD-EPI equation **(A)** and urine protein to creatinine ratios (UPCR; **B**). Line colors indicate time periods for each patient with native kidney, dialysis, kidney graft, as illustrated by the legend to the right. X-axis shows the time in months from treatment begin with eculizumab. For patients not receiving eculizumab, the first date of visit is timepoint zero. Y-axis is eGFR (ml/min/1.73m^2; **A**), and UPCR (g/g; **B**). Tick labels in **(A)** indicate chronic kidney disease stages, as defined by KDIGO [1]. Each facet illustrates one patient. Horizontal colored boxes below each facet indicate treatment intervals for other immunosuppressive drugs. Color labels for each drug are indicated in the legend to the right. Patients receiving standard of care (SoC) treatment, or eculizumab are indicated.
Abbreviations: ffp, fresh-frozen plasma; ivig, intravenous immunoglobins; mmf, mycophenolate mofetil; pex, plasma exchange; SoC, standard of care treatment.

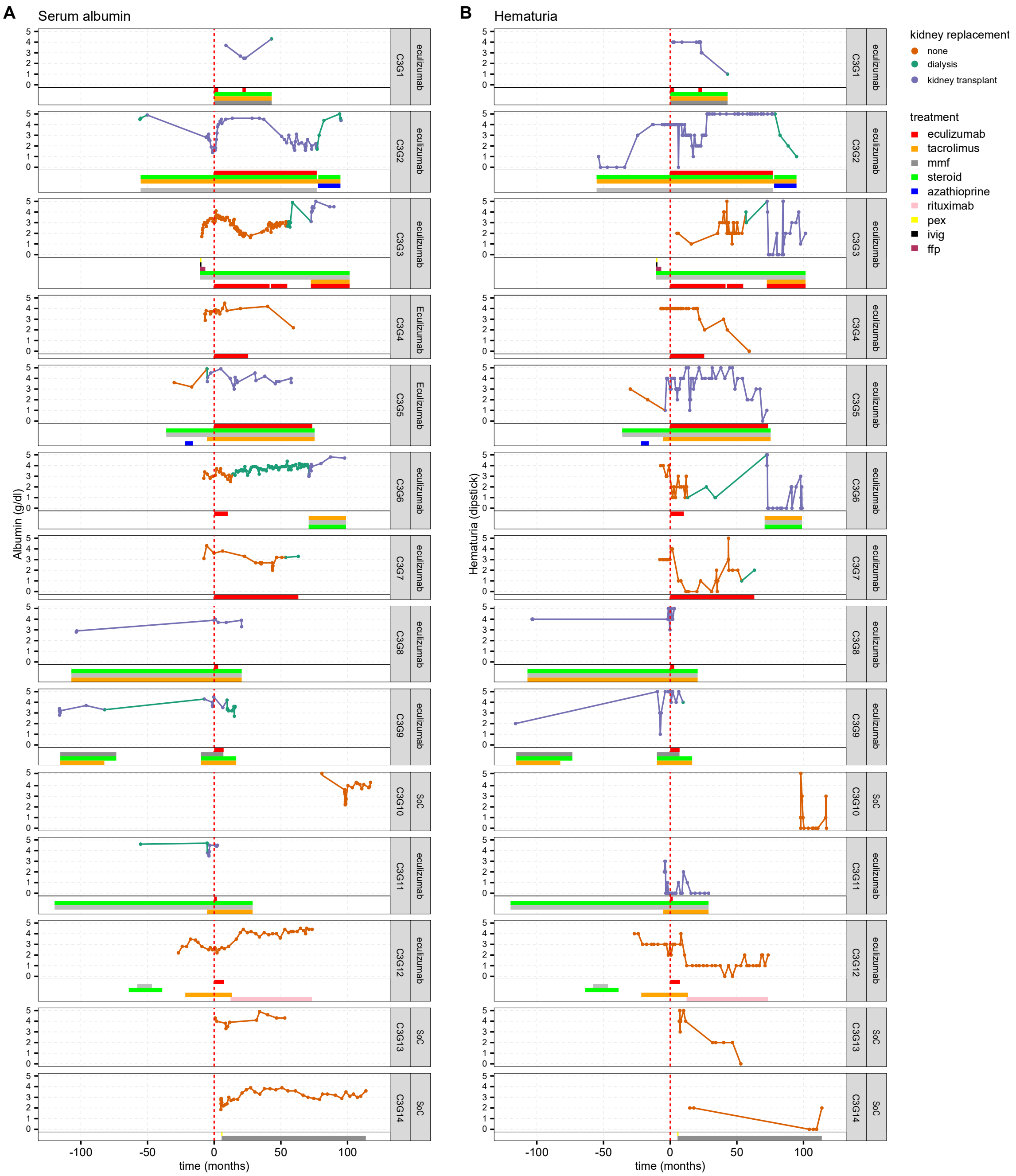

Supplementary Figure S2. Course of serum albumin and urine blood for individual cases.

Line graphs indicating serum albumin **(A)** and hematuria quantified by dipstick (scale 0 – 5; **B**). Line colors indicate time periods for each patient with native kidney, dialysis, kidney graft, as indicated by the legend to the right. X-axis shows the time in months from treatment begin with eculizumab. For patients not receiving eculizumab, the first date of visit is timepoint zero. Y-axis is serum albumin (g/dl; **A**), and hematuria (**B**). Each facet signifies one patient. Horizontal colored boxes below each facet indicate treatment intervals for immunosuppressive drugs. Color labels for each drug are indicated in the legend to the right. Patients receiving standard of care (SoC) treatment, or eculizumab are indicated.

Abbreviations: ffp, fresh-frozen plasma; ivig, intravenous immunoglobins; mmf, mycophenolate mofetil; pex, plasma exchange; SoC, standard of care treatment.

References to Supplementary Material

1. Levin A, Stevens PE, Bilous RW, Coresh J, De Francisco AL, De Jong PE, Griffith KE, Hemmelgarn BR, Iseki K, Lamb EJ: **Kidney Disease: Improving Global Outcomes (KDIGO) CKD Work Group. KDIGO 2012 clinical practice guideline for the evaluation and management of chronic kidney disease.** *Kidney international supplements* 2013, **3**(1):1-150.
